# Dramatic increase in SHP2 binding activity of *Helicobacter pylori* Western CagA by EPIYA-C duplication: its implications in gastric carcinogenesis

**DOI:** 10.1038/srep15749

**Published:** 2015-10-28

**Authors:** Lisa Nagase, Takeru Hayashi, Toshiya Senda, Masanori Hatakeyama

**Affiliations:** 1 Division of Microbiology, Graduate School of Medicine, The University of Tokyo, Tokyo 113-0033, Japan; 2 CREST, Japan Science and Technology Agency, Saitama 332-0012, Japan; 3 Structural Biology Research Center, Institute of Materials Structure Science, High Energy Accelerator Research Organization (KEK), Tsukuba 305-0801, Japan

## Abstract

Infection with *cagA*-positive *Helicobacter pylori* is critically associated with the development of gastric cancer. The *cagA*-encoded CagA is delivered into gastric epithelial cells via type IV secretion, where it interacts with and thereby deregulates the pro-oncogenic phosphatase SHP2. East Asian CagA and Western CagA are two major CagA species produced by *H. pylori* circulating in East Asian countries and in the rest of the world, respectively. The SHP2 binding site of Western CagA, termed the EPIYA-C segment, variably duplicates and infection with *H. pylori* carrying Western CagA with multiple EPIYA-C segments is a distinct risk factor of gastric cancer. Here we show that duplication of EPIYA-C from one to two or more increases SHP2 binding of Western CagA by more than one hundredfold. Based on the decisive difference in SHP2 binding, Western CagA can be divided into two types: type I CagA carrying a single EPIYA-C segment and type II CagA carrying multiple EPIYA-C segments. Gastric epithelial cells expressing type II CagA acquire the ability to invade extracellular matrices, a malignant cellular trait associated with deregulated SHP2. A big leap in SHP2 binding activity may therefore provide molecular basis that makes type II Western CagA a distinct gastric cancer risk.

Chronic infection with *Helicobacter cagA*-positive strains is causally associated with the development of gastric carcinoma^1^,^2^, the third most common cause of death from cancer worldwide^3^. The *cagA* gene-encoded CagA protein is delivered into gastric epithelial cells via the bacterial type IV secretion system. Once inside the host cell, CagA is tethered to the inner plasma membrane, where it undergoes tyrosine phosphorylation by Src family kinases or c-Abl kinase at the Glu-Pro-Ile-Tyr-Ala (EPIYA) motifs that are present in a variable number in the disordered C-terminal region of CagA^4^,^5^,^6^,^7^. Based on sequences flanking each of the EPIYA motifs, four distinct EPIYA segments, termed EPIYA-A, -B, -C and -D, have been determined^8^,^9^. Difference in the arrangement of these EPIYA segments in the C-terminal region makes it possible to classify the *H. pylori* effector into Western CagA and East Asian CagA, which are characterized by the presence of EPIYA-C and EPIYA-D segments, respectively. *H. pylori* strains carrying East Asian CagA are predominant in Japan, Korea, and China, whereas *H. pylori* strains carrying Western CagA are distributed worldwide except for East Asian countries. A unique feature associated with the EPIYA-C segment of Western CagA is that it tandemly duplicates with a variable number, mostly from one to three but can extend up to six as reported to date^10^. The frequencies of Western CagA containing one, two, and three EPIYA-C segments are approximately 60–70%, 20–30%, and <5%, respectively^10^,^11^. In contrast to EPIYA-C, the EPIYA-D segment hardly duplicates in East Asian CagA species. Upon tyrosine phosphorylation in gastric epithelial cells, the EPIYA-C segment or EPYA-D segment of CagA serves as the docking site for the SH2 domain-containing protein tyrosine phosphatase SHP2^12^, a *bona-fide* oncoprotein mutated in a variety of human malignancies^13^. SHP2 binds to CagA via the N-terminally located tandem SH2 domains (N-SH2 and C-SH2), both of which recognize tyrosine-phosphorylated peptides with similar specificities. East Asian CagA containing a single EPIYA-D segment binds to SHP2 more strongly than does Western CagA containing a single EPIYA-C segment^9^. Among Western CagA species, those containing a larger number of EPIYA-C segments undergo stronger tyrosine phosphorylation and exhibit greater SHP2 binding activity than do those having less EPIYA-C segments. Because CagA-SHP2 interaction deregulates the pro-oncogenic SHP2 phosphatase, the complex formation has been considered to play an important role in gastric carcinogenesis^8^. In fact, transgenic mice systemically expressing CagA spontaneously develop gastrointestinal cancers and hematological malignancies in an EPIYA-dependent manner, arguing for an important role of CagA-SHP2 interaction in *in vivo* tumorigenesis^14^. Furthermore, the results of a number of recent clinico-epidemiological studies have shown that infection with *H. pylori* strains carrying Western CagA with two or more EPIYA-C segments is a greater risk for the development of gastric carcinoma than is infection with *H. pylori* carrying CagA with a single EPIYA-C segment^15^,^16^,^17^,^18^,^19^,^20^.

Since SHP2 binding is the only known CagA activity for which the magnitude is correlated with the number of EPIYA-C segments^8^,^9^, the degree of CagA-SHP2 interaction may link the number of EPIYA-C segments with gastric cancer risk. In this work, we carried out a quantitative study for the CagA-SHP2 interaction and found that the strength of CagA-SHP2 binding was dramatically elevated by more than a hundredfold upon duplication of the CagA EPIYA-C segment. The robust increase in SHP2 binding activity of CagA by EPIYA-C duplication was also associated with the marked enhancement of cell invasion phenotype into the extracellular matrix, a malignant cellular trait associated with SHP2 deregulation^21^,^22^,^23^,^24^. These observations provide a molecular basis underlying the clinico-epidemiological observation that Western CagA with multiple EPIYA-C segments is a distinct risk factor for the development of gastric cancer.

## Results

### Increase in the number of EPIYA-C segments potentiates CagA-SHP2 interaction *in vitro*

**Figure 1:**
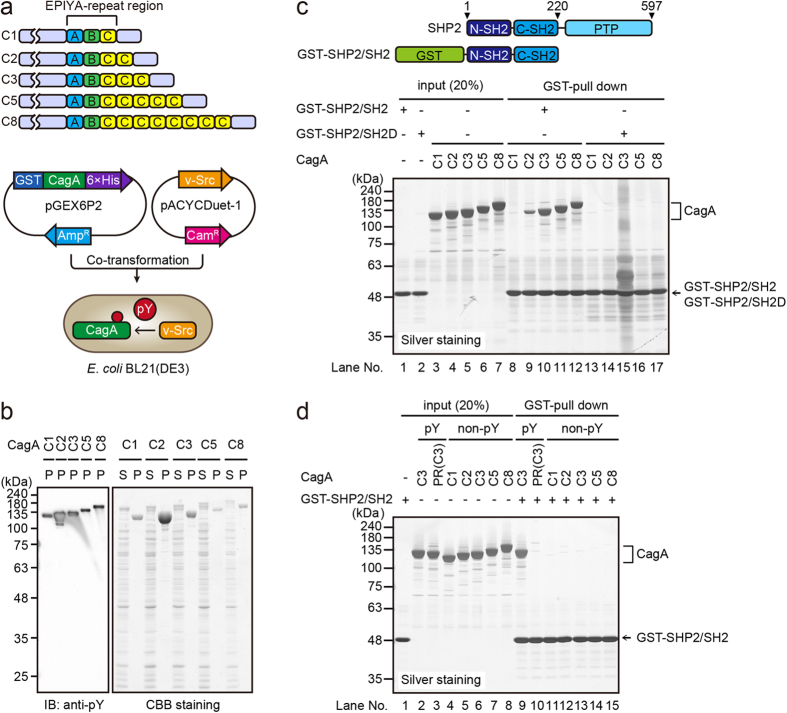
Tyrosine phosphorylation-dependent *in vitro* interaction of CagA with SHP2. (**a**) Schematic representation of *H. pylori* CagA variants used in this work (upper). Strategy for tyrosine phosphorylation of recombinant CagA in *E. coli* (lower). *E. coli* BL21(DE3) was co-transformed with a GST-fused CagA expression vector and a v-Src expression vector. (**b**) Recombinant CagA proteins [CagA(Cn)-His] containing various numbers of EPIYA-C segments (C1, C2, C3, C5, and C8) were purified from *E. coli* co-expressing v-Src and subjected to immunoblotting with an anti-phosphotyrosine (pY) antibody (left) or CBB staining (right). S: supernatant of *E. coli* lysates containing GST-fused CagA, P: purified CagA after the cleavage of GST-tag. (**c**) GST pull-down assay was performed using tyrosine-phosphorylated CagA shown in (**b**) and GST-fused N-terminal SHP2 fragment containing two tandem-repeated SH2 domains, either functionally active (GST-SHP2/SH2) or dead (GST-SHP2/SH2D). CagA bound to GST-SHP2/SH2 was detected by silver staining. (**d**) GST pull-down assay was performed using GST-SHP2/SH2 and CagA containing three EPIYA-C segments (C3) or PR-CagA purified from *E. coli* expressing v-Src (lanes 1–3, 9, 10). GST pull-down assay was also performed using GST-SHP2/SH2 and CagA proteins containing various numbers of the EPIYA-C segments (C1, C2, C3, C5, C8) purified from *E. coli* without v-Src expression (lanes 4–8, 11–15). CagA bound to GST-SHP2/SH2 was detected by silver staining.

We previously reported that an increase in the number of EPIYA-C segments enhances complex formation of Western CagA with the pro-oncogenic phosphatase SHP2 in gastric epithelial cells^8^,^9^. Since the EPIYA-C segment number has also been shown to be associated with gastric cancer risk^15^,^16^,^17^,^18^,^19^,^20^, we sought to quantitatively determine the relationship between number of EPIYA-C segments and SHP2 binding activity of CagA by reconstituting the CagA-SHP2 interaction *in vitro*. Since tyrosine phosphorylation of the EPIYA-C segment is an essential prerequisite for the interaction of Western CagA with SHP2, we needed to tyrosine-phosphorylate CagA for the *in vitro* binding study. Previously, we established a CagA expression system in *Escherichia coli* and succeeded in purifying the full-length recombinant CagA protein in a high yield^5^. We therefore utilized this bacterial system for expressing and purifying tyrosine-phosphorylated CagA. To this end, we first constructed an *E. coli* expression vector for the N-terminal GST-fused and C-terminal His-tagged Western CagA protein, which contains a variable number of functional EPIYA-C segments (1, 2, 3, 5, or 8), from the pGEX6P2 vector carrying an ampicillin-resistance gene (Fig. 1a). In this construct, the EPIYA motifs in the EPIYA-A and EPIYA-B segments were substituted to the EPIFA sequence to prevent tyrosine phosphorylation. The vector-derived GST-CagA fusion protein was designated GST-CagA(Cn)-His, where n indicated the number of EPIYA-C segments. In Western CagA reported to date, the number of EPIYA-C duplications can be extended up to 6 times^10^. The reason why we made CagA(C8), which may be naturally absent, was to deduce a biochemical law that associates the number of EPIYA-C segments with SHP2-binding strength by providing more data. We also constructed an *E. coli* expression vector for v-Src from the pACYCDuet1 vector, which carries a chloramphenicol-resistance gene. The *E. coli* BL21(DE3) strain was then co-transformed by the GST-CagA(Cn)-His and v-Src vectors and was selected in bacterial media containing ampicillin and chloramphenicol (Fig. 1a). GST-CagA(Cn)-His proteins were purified from the bacterial lysates by affinity chromatography using Ni-NTA agarose beads and glutathione sepharose beads, followed by treatment with PreScission to cleave the GST-tag. The purified CagA(Cn)-His proteins were then separated on sodium dodecyl sulfate-polyacrylamide gel electrophoresis (SDS-PAGE) and subjected to immunoblotting with an anti-phosphotyrosine antibody or Coomassie Brilliant Blue (CBB) staining. As shown in Fig. 1b, all of the CagA(Cn)-His proteins were highly purified as well as tyrosine-phosphorylated [hereafter designated as “pY-CagA(Cn)-His”]. In contrast, CagA expressed in *E. coli* without v-Src was not tyrosine-phosphorylated, indicating CagA tyrosine phosphorylation was strictly dependent on co-expressed v-Src (Fig. S1). To determine the extent of v-Src-mediated tyrosine phosphorylation on the pY-CagA(Cn)-His proteins, we made use of the phosphate-affinity SDS-PAGE, termed Phos-tag SDS-PAGE, which can detect differentially phosphorylated protein isoforms as distinctly migrating bands^25^. The results of the Phos-tag experiment using CagA(C3)-His or CagA(C8)-His revealed that co-expression of v-Src gave rise to a single CagA band, which migrated more slowly than the non-phosphorylated form due to multi-site tyrosine phosphorylation (Fig. S2). Thus, each of CagA(Cn)-His proteins was almost uniformly tyrosine-phosphorylated by v-Src in *E. coli.* Next, to evaluate the extent of EPIYA-C tyrosine phosphorylation in pY-CagA(Cn)-His, we utilized a phosphorylation-resistant (PR) CagA(C3)-His, which had been made from ABCCC-type CagA by replacing all of the EPIYA-motifs with the EPIFA sequences and thus did not undergo tyrosine phosphorylation in mammalian cells^9^,^12^. We constructed a pGEX6P2-derived GST-PR-CagA(C3)-His vector and expressed it together with v-Src in *E. coli*. After purification and cleavage of the GST-tag, PR-CagA(C3)-His was obtained. The level of tyrosine phosphorylation of PR-CagA(C3)-His by v-Src was then compared with that of CagA(C3)-His by v-Src. As shown in Fig. S3, PR-CagA(C3)-His was substantially less phosphorylated than CagA(C3)-His, showing that the EPIYA-C segments were major sites of tyrosine phosphorylation by v-Src in *E. coli* while indicating that v-Src also phosphorylated CagA on tyrosine residues that are distinct from those present in the EPIYA-C segments.

Next, using purified pY-CagA(Cn)-His and a GST-tagged N-terminal SHP2 fragment containing the two SH2 domains (GST-SHP2/SH2, Fig. 1c), we performed a GST pull-down assay. The results of the experiment revealed that GST-SHP2/SH2 pulled down all of the pY-CagA(Cn)-His proteins (Fig. 1c, lanes 8–12). In contrast, GST-SHP2/SH2 failed to pull down CagA(Cn)-His, which had been prepared from *E. coli* without co-expressing v-Src and thus did not undergo tyrosine phosphorylation (Fig. 1d, lanes 11–15, and Fig. S1). Likewise, functional inactivation of both of the SH2 domains in GST-SHP2/SH2 resulted in the loss of pY-CagA(Cn)-His binding (Fig. 1c, lanes 13–17). In this regard, since pY-CagA(Cn)-His contained phosphotyrosine residues other than those constituting the EPIYA motifs (Fig. S3), there remained the possibility that GST-SHP2/SH2 formed a complex with CagA via phosphotyrosine residues distinct from those constituting the EPIYA-C segments. To exclude this possibility, we carried out a pull-down assay using GST-SHP2/SH2 and tyrosine-phosphorylated PR-CagA(C3)-His [pY-PR-CagA(C3)-His] and found that they did not bind to each other (Fig. 1d, lane 10). The result provided evidence that the interaction of CagA with the SHP2 SH2 domains is strictly dependent on the tyrosine-phosphorylated EPIYA-C segment. From these observations, we concluded that the complex formation between Western CagA and SHP2 was faithfully reconstituted *in vitro* using recombinant proteins. Also, the results of the pull-down experiment were consistent with the results of previous studies showing that the degree of CagA-SHP2 complex formation was enhanced as the number of EPIYA-C segments increased^9^. Notably, there was a marked increase in the amount of pulled-down CagA when the number of EPIYA-C segments increased from one (C1) to two (C2) (Fig. 1c, lanes 8 and 9). The amount of pulled-down CagA was also increased as the number of EPIYA-C segments increased from two (C2) to three (C3). However, the amount of pulled-down CagA appeared to be saturated when the number of the EPIYA-C segments became three (C3) or more in this experimental setting (Fig. 1c, lanes 10–12 and Fig. S4).

### EPIYA-C duplication confers a non-linear increase in the SHP2 binding affinity of CagA

**Figure 2:**
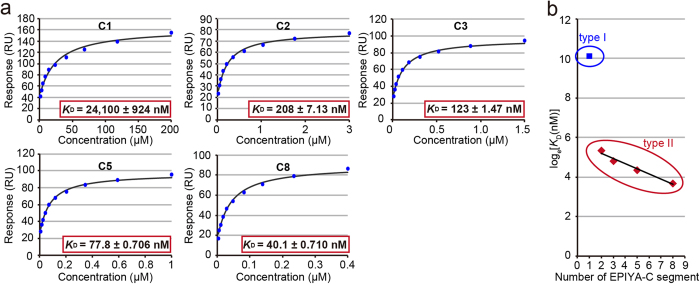
Quantitative study of CagA-SHP2 interaction. (**a**) Surface plasmon resonance (SPR) analysis was performed using tyrosine-phosphorylated CagA containing a variable number of EPIYA-C segments (C1, C2, C3, C5, and C8) and the N-terminal SHP2 fragment containing two SH2 domains (SHP2/SH2). Equilibrium dissociation constant (*K*_D_) of CagA-SHP2 complex was determined by a Scatchard plot, which indicates the amount of bound at each concentration of SHP2/SH2 (Mean ± SEM, n = 3). (**b**) The logarithmic value of *K*_D_ in each number of EPIYA-C segments was plotted and regression line was calculated. A blue spot means *K*_D_ of binding of SHP2/SH2 to CagA with a single EPIYA-C segment (type I Western CagA, circled in blue) whereas red spots represent *K*_D_ values of binding of SHP2/SH2 to CagA with multiple EPIYA-C segments (type II Western CagA, circled in red).

The results of the GST pull-down assay raised the possibility that duplication of the EPIYA-C segment dramatically enhances complex formation between CagA and SHP2. However, the GST pull-down experiment *per se* was not highly quantitative in describing protein-protein interaction, only providing a snapshot of CagA-SHP2 complex formation, the degree of which is determined by relative concentrations of GST-SHP2/SH2 and recombinant CagA in the reaction mixture. We therefore sought to quantitatively determine the SHP2 binding activity of CagA carrying a variable number of EPIYA-C segments. To this end, binding strength of the CagA-SHP2 complex was directly measured by using the surface plasmon resonance (SPR) technique. Specifically, recombinant pY-CagA(Cn)-His was immobilized on a sensor chip as the ligand and recombinant SHP2/SH2 was added to the flow cell as the analyte. The equilibrium dissociation constant (*K*_D_) of their interaction was calculated by Scatchard plot analysis (Fig. 2a). The results of SPR analysis revealed that binding strength of between pY-CagA(Cn)-His and SHP2/SH2 interaction becomes greater as the repeat number of EPIYA-C segments increases; the *K*_D_ values determined in three independent measurements were 24,100 ± 924 nM for CagA(C1), 208 ± 7.13 nM for CagA(C2), 123 ± 1.47 nM for CagA(C3), 77.8 ± 0.706 nM for CagA(C5), and 40.1 ± 0.710 nM for CagA(C8). Of particular note was the two orders of magnitude increase in SHP2/SH2-binding strength from CagA(C1) to CagA(C2). In contrast, increases in SHP2/SH2-binding strength from CagA(C2) to CagA(C3), CagA(C3) to CagA(C5), and CagA(C5) to CagA(C8) were all less than twofold. The results of the SPR analysis were reasonably consistent with the results of the GST pull-down experiment (Fig. 1c and Fig. S4) because the concentration of CagA (50 nM) in the pull-down assay was closer to the SHP2/SH2 binding affinities (*K*_D_ values) of CagA(C3) (123 nM), CagA(C5) (77.8 nM), and CagA(C8) (40.1 nM) than those of CagA(C2) (208 nM) and CagA(C1) (24.1 μM). SHP2/SH2 used in the SPR analysis lacked the C-terminal phosphatase domain of SHP2. Based on the crystal structure of the entire SHP2 molecule, the C-terminal phosphatase domain is positioned opposite to the site of the clefts of the two SH2 domains, to which tyrosine-phosphorylated proteins bind^26^. Accordingly, the lack the C-terminal phosphatase domain did not seem to substantially influence the CagA-SHP2 interaction. In fact, SPR analysis demonstrated that binding of pY-CagA(C2)-His with full-length SHP2 (369 ± 87 nM) and that of pY-CagA(C2)-His with SHP2/SH2 (208 ± 7.13 nM) were comparable (Fig. S5). Thus, interaction of CagA with SHP2/SH2 recapitulated interaction of CagA with full-length SHP2.

Using the binding data, we created a single logarithmic graph, with *K*_D_ converted into the logarithmic value plotted on the y-axis and the number of EPIYA-C segments plotted on the x-axis (Fig. 2b). In cases of CagA containing two or more EPIYA-C segments, the *K*_D_ values of the CagA-SHP2/SH2 interaction were fitted to a linear regression line on a semi-log scale (y = 305.38e^−0.261x^; where X means the number of EPIYA-C segments and Y means *K*_D_). However, the *K*_D_ value of the CagA(C1)-SHP2/SH2 interaction was completely deviated from the regression line, indicating that the SHP2 binding affinity of CagA with a single EPIYA segment was extremely low compared to that of CagA containing two or more EPIYA-C segments. The decisive difference in SHP2 binding activity made it possible to divide Western CagA into two distinct types: type I Western CagA, which was characterized by a single EPIYA-C segment (circled in blue in Fig. 2b), and type II Western CagA, which was characterized by multiple EPIYA-C segments (circled in red in Fig. 2b).

### Biologically relevant interaction of CagA with SHP2 requires multiple EPIYA-C segments

**Figure 3:**
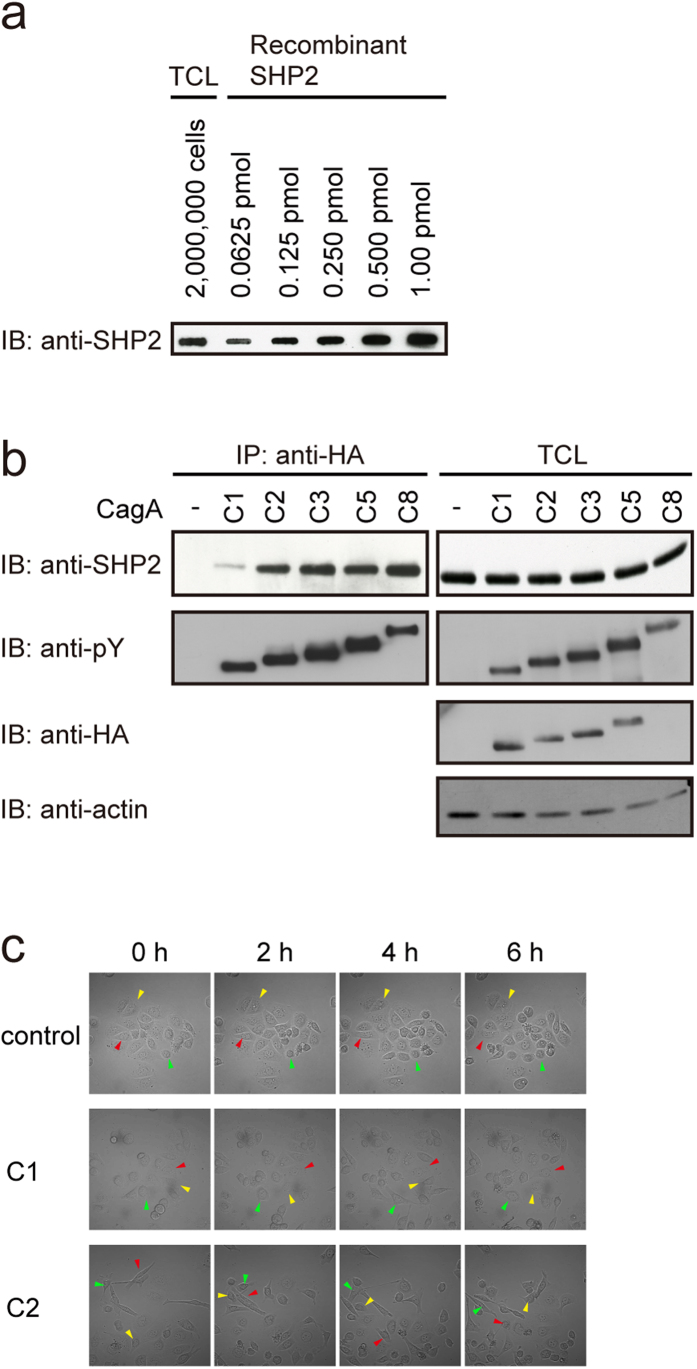
Complex formation between SHP2 and CagA containing a variable number of EPIYA-C segments in gastric epithelial cells. (**a**) Total cell lysates (TCLs) prepared from AGS gastric epithelial cells (2 million cells) and a series of diluted recombinant SHP2 samples were subjected to an immuno-dot blot assay with an anti-SHP2 antibody. The intracellular concentration of SHP2 in AGS cells was calculated by quantifying intensities of each SHP2 bands with a luminescence image analyzer. (**b**) AGS cells were transiently transfected with an expression vector for an HA-tagged CagA containing a variable number of EPIYA-C segments (C1, C2, C3, C5, or C8). At 24 h after transfection, TCLs were prepared and subjected to immunoprecipitation with an anti-HA antibody. The anti-HA immunoprecipitates (IP) and TCLs were subjected to immunoblotting (IB) with the indicated antibodies. (**c**) AGS cells were transfected with an expression vector for CagA-ABC (C1) or CagA-ABCC (C2). At 12 h after transfection as a starting point for time-lapse imaging, cell migration were observed at 3-min intervals for 6 hours by differential interference contrast microscopy. Images every 2 hours were shown. Arrowheads of the same color (red, yellow, or green) indicate the positions of the same cell during the time course. See also S1–S3 Movies.

To gain an insight into the biological relevance of the observed differences in the *K*_D_ value of CagA-SHP2 interaction according to the number of EPIYA-C segments, we sought to determine the concentration of SHP2 in gastric epithelial cells. To this end, we carried out a quantitative anti-SHP2 immuno-dot blot assay of whole cell lysates prepared from AGS human gastric epithelial cells using recombinant SHP2 as a marker for concentration determination (Fig. 3a). Since the average volume of epithelial cells is approximately 4000 μm^3^, intracellular concentration of SHP2 was estimated to be about 100 nM. Assuming that the local concentration of *H. pylori*-injected CagA in gastric epithelial cells could reach up to 1 μM (10-fold greater than the estimated concentration of endogenous SHP2), less than 5% of SHP2 binds to type I Western CagA, whereas more than 80% of SHP2 could associate with type II Western CagA at that CagA concentration (1 μM). Stochastic interaction of SHP2 with type I CagA may therefore require an extremely high concentration of CagA in the injected host cells. In contrast, the presence of multiple EPIYA-C segments in type II CagA guarantees CagA-SHP2 complex formation to a level that is sufficient to induce pathogenic action with a much smaller amount of injected CagA.

To test this idea, we transiently transfected expression vectors for Western CagA proteins containing different numbers of EPIYA-C segments in AGS cells. In the transient transfection experiment in which transfection efficiencies were comparable among distinct CagA expression vectors, ectopically expressed CagA was a major tyrosine-phosphorylated protein in AGS cells (Fig. S6). In this experiment, we repeatedly observed reduced expression of CagA, both at the protein level and in the number of CagA-positive cells, as the number of EPIYA-C segments increased (Fig. 3b and Fig. S7). Since the anti-HA blotting and anti-phosphotyrosine (pY) blotting of total cell lysates (TCLs) prepared from AGS cells (Fig. 3b, right panels) were carried out by reprobing the same filter, the anti-pY antibody (4G10) was substantially more sensitive than the anti-HA (3F10) antibody in detecting CagA (especially when CagA contains multiple EPIYA-C segments). As a consequence, HA-tagged CagA(C8), which contains 8 × EPIYA-C tyrosine phosphorylation sites, could only be visualized by anti-pY immunoblotting. The reduced CagA expression may be due to increased instability or elevated toxicity of Western CagA carrying a larger number of EPIYA-C segments in AGS cells. Despite reduced CagA expression in cells, however, the amount of endogenous SHP2 bound to CagA became greater as the EPIYA-C repeat number increased. Here, again, a decisive difference in SHP2 binding was observed between type I Western CagA and type II Western CagA, reconfirming the results of the *in vitro* binding experiment despite the previous observation that interaction of SHP2 with CagA containing a single EPIYA-C segment is enhanced by PAR1-mediated CagA dimerization in cells^27^,^28^. Since the amount of CagA expressed in AGS cells by the transient transfection experiment was comparable to that of CagA delivered into AGS cells upon *in vitro* infection with *cagA*-positive *H. pylori* (MOI = 100) as determined by the levels of tyrosine-phosphorylated CagA (Fig. S8), such a big difference in CagA-SHP2 complex formation between type I Western CagA and type II Western CagA may also occur in human gastric mucosa infected with Western *cagA-*positive *H. pylori* strains.

Deregulation of SHP2 by CagA in AGS cells induces an elongated cell shape known as the hummingbird phenotype while increasing cell motility^29^. We therefore performed time-lapse microscopic analysis of AGS cells transiently transfected with an expression vector for CagA containing a various number of EPIYA-C segments (Fig. 3c). In this experiment, again, the expression level of transfected CagA in AGS cells was inversely correlated with the number of EPIYA-C segments. However, cells transfected with a vector for type II Western CagA exhibited elevated cell motility that was far greater than that of cells transfected with a vector for type I Western CagA (Fig. 3c and Movies S1–S3). Thus, duplication of the EPIYA-C segment dramatically boosted the motogenetic activity of CagA.

### Cell invasion phenotype by CagA requires multiple EPIYA-C segments

**Figure 4:**
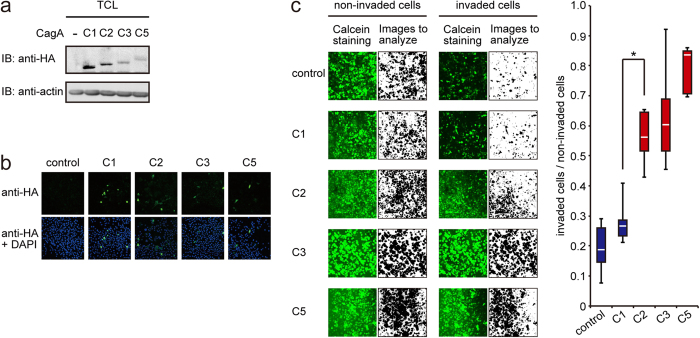
Invasion assay of gastric epithelial cells expressing CagA with a variable number of EPIYA-C segments. AGS cells transiently transfected with an expression vector for an HA-tagged CagA containing a variable number of EPIYA-C segments (C1, C2, C3, or C5) were subjected to invasion assay. (**a**) Expression of each CagA proteins in transfected cells was determined by immunoblotting. (**b**) Expression of each CagA proteins in transfected cells was examined by immunostaining using an anti-HA antibody (green). Nuclei were also stained with DAPI (blue). (**c**) Cells were stained by Calcein and the invading cell population and non-invading cell population were quantified using ImageJ software (black images for Calcein-stained cells) (left). The magnitudes of strength of invasion phenotype were determined by evaluating relative ratio of cells that passed through the filter to invade into collagen gel matrices (invaded cells) in regard to cells that didn’t pass through the filter (non-invaded cells) and are shown as box plots (right). *P < 0.01, Mann-Whitney *u* test.

SHP2 is a *bona-fide* oncoprotein, gain-of-function mutations of which have been found in various human malignancies^13^. Deregulated SHP2 aberrantly activates the Erk signaling pathway by both Ras-dependent and -independent mechanisms^13^. SHP2 also promotes cell migration and stimulates cell invasion^21^,^22^,^23^,^24^. These SHP2 activities may play important roles in conferring malignant traits on cancer-predisposed cells. To gain an insight into the pathophysiological relevance of SHP2 deregulation by CagA, especially CagA containing multiple EPIYA-C segments, in oncogenesis, we carried out a cell invasion assay using collagen matrix gel. Consistent with the above-described observation, the expression level of transfected CagA in AGS cells was decreased as the number of EPIYA-C segments increased (Fig. 4a,b). A striking observation was that there was a huge difference in invasion potential between AGS cells transfected with a vector for type I CagA and AGS cells transfected with a vector for type II CagA (Fig. 4c). This finding indicated that the presence of multiple EPIYA-C segments in CagA bestows a strong invasive phenotype on gastric epithelial cells. Since cell invasion into surrounding tissue and spread of cells to distant sites are important traits of neoplastic cells^30^, duplication of the EPIYA-C segment is considered to be a critical structural determinant for the oncogenic potential of Western CagA.

## Discussion

The EPIYA-C segment is unique to Western CagA and is characterized by its variable duplications, mostly between one and three, among Western CagA species^8^,^9^,^11^. Physical interaction of Western CagA with SHP2 is mediated exclusively through the tyrosine-phosphorylated EPIYA-C segment of CagA and one or both of the two SH2 domains of SHP2 that display similar recognition specificities^9^. A number of clinico-epidemiological studies have shown that *H. pylori* strains harboring two or more CagA EPIYA-C segments are associated with gastric cancer^15^,^16^,^17^,^18^,^19^,^20^, although several studies failed to show a clear association between the number of EPIYA-C segments and gastric cancer, most probably due to small sample sizes^31^,^32^. The number of EPIYA-segments may therefore substantially influence the oncogenic potential of Western CagA. The present study is the first comprehensive and quantitative study for the role of EPIYA-C repeat number in the CagA action, revealing that duplication of EPIYA-C segments increases SHP2 binding activity of Western CagA by more than a hundredfold. Given the decisive difference in SHP2 binding activity, Western CagA can be divided into two types: type I Western CagA, which is characterized by the presence of a single EPIYA-C segment and by an extremely weak SHP2 binding activity, and type II Western CagA, which is characterized by the presence of multiple EPIYA-C segments and by a strong SHP2 binding activity.

**Figure 5:**
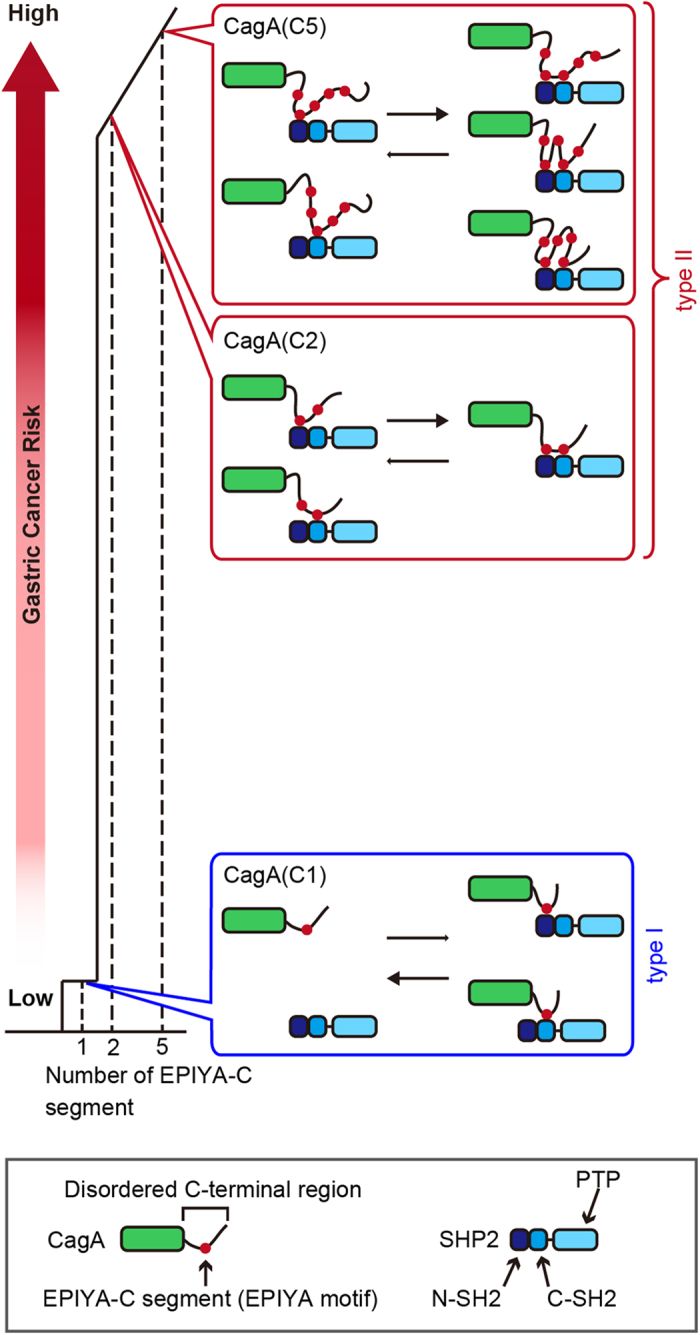
A mechanism that correlates the number of the CagA EPIYA-C segments with gastric cancer risk. CagA containing multiple EPIYA-C segments (type II Western CagA) binds to either the N-SH2 or C-SH2 domain of SHP2 and subsequently the second binding to the other SH2 domain occurs, thereby forming a stable bivalent complex. On the other hand, CagA containing a single EPIYA-C segment (type I Western CagA) cannot make the second interaction, leaving the monovalent CagA-SHP2 complex unstable. The strength of complex formation steadily elevates among type II CagA as the number of the EPIYA-C segments increases from two to more, due to an elevated local concentration of EPIYA-C. The difference between a single EPIYA-C segment and two or more EPIYA-C segments in the SHP2 binding activity provides the molecular basis underlying an increased risk of gastric cancer in individuals infected with *H. pylori* carrying type II Western CagA.

The big leap in SHP2 binding of Western CagA through EPIYA-C duplication is mechanistically explained as follows. In the case of CagA containing a single EPIYA-C segment, monovalent interaction of EPIYA-C with either the N-SH2 or C-SH2 domain of SHP2 determines the strength of complex formation. Upon duplication of the EPIYA-C segment from one to two, the CagA-SHP2 interaction becomes bivalent. This process should be initiated via binding of one of the two SHP2 SH2 domains to an EPIYA-C segment. The monovalent interaction brings the resting EPIYA-C segment in close proximity to the other SH2 domain and thereby promotes the second interaction. Even after a stable bivalent complex is formed, one of the two bonds should eventually be lost depending on its association-dissociation equilibrium. However, the remaining monovalent interaction greatly increases the chance of re-association of the dissociated sites in the complex. Additional expansion the EPIYA-C segments from two to three or more gives rise to a stepwise but much more moderate increase (less than twofold per one EPIYA-C expansion) in the strength of CagA-SHP2 interaction. This gentle but consistent increase in binding strength is rather difficult to understand if the expanded EPIYA-C segments constitute a solid structure. Accordingly, we consider that the intrinsically disordered nature of the C-terminal CagA region spanning the EPIYA segments^5^,^33^ is crucial for the observed increase in SHP2 binding activity according to the number of EPIYA-C segments. In CagA containing three or more EPIYA-C segments, each of the segments can move without obvious structural constraints because of the intrinsic disorder and thus has ready access to one of the two SH2 domains of SHP2 in the proximate state. Here, again, interactions between multiple EPIYA-C segments and SHP2 should occur in a sequential manner. First, one of the two SHP2 SH2 domains binds to an EPIYA-C segment, which increases the local concentration of the EPIYA-C segments that flop around the first binding site by restricting its three-dimensional diffusion, and thereby enhances binding to the second SH2 domain (Fig. 5). Whereas SHP2 binding of CagA containing a single EPIYA-C segment (type I CagA) could be stabilized to a certain extent through PAR1-mediated CagA dimerization as previously reported^27^,^28^, it is highly unlikely that such a weak CagA-SHP2 interaction (*K*_D_ > 20 μM) can easily deregulate a biologically meaningful fraction of endogenous SHP2, the concentration of which is estimated to be approximately 100 nM in AGS gastric epithelial cells. On the other hand, the *K*_D_ values between SHP2 and CagA containing two or more EPIYA-C segments (type II CagA) are roughly equal to or less than the endogenous concentration of SHP2, indicating that type II is capable of stochastically interacting with endogenous SHP2 in the injected host gastric epithelial cells.

SHP2, the binding target of the EPIYA-C segment, is the pro-oncogenic phosphatase critically required for full activation of the Ras-Erk MAP kinase pathway. Deregulated phosphatase activity of SHP2 is also crucial in the cell invasion phenotype^21^,^22^,^23^,^24^, and CagA has been reported to stimulate cell invasion via SHP2 deregulation^34^. Aberrant migration of epithelial cells through extracellular matrices represents invasion and metastasis of neoplastic cells^35^,^36^. The present study revealed that the degree of cell-invasive phenotype induced by CagA faithfully reflects the strength of CagA-SHP2 interaction, showing dramatic differences between type I Western CagA and type II Western CagA. Enhanced migration and invasion of CagA-injected gastric epithelial cells may disorganize the epithelial monolayer as well as extracellular matrices and thereby deteriorate *H. pylori*-induced inflammation and inflammation-promoted gastric carcinogenesis while providing a microenvironmental milieu that fosters invasion and metastasis of neoplastic cells.

The association of tandem-repeat polymorphisms in a protein and clinical outcomes has been reported in several human disorders. Polyglutamine (PolyQ) diseases are inherited neurological disorders elicited by the abnormal expansion of glutamine-encoding CAG triplet repeats within endogenous genes^37^. The diseases include Huntington’s disease, spino-bulbar muscular atrophy (SBMA) and spinocerebellar ataxia (SCA) and are suspected to be due to length-dependent toxicity of an expanded polyQ stretch. Another example is the prion protein (PrP), which induces prion diseases, lethal transmissible neurodegenerative disorders that include Creutzfeldt-Jakob disease (CJD). PrP contains five tandem repeats of an octapeptide, which may bind to divalent cations such as copper or zinc. An increase of the repeat number to six or more or a decrease to three is linked to hereditary CJD^38^, indicating that the number of octapeptide repeats influences the conversion process of the cellular isoform of PrP (PrP^C^) into an abnormal isoform (PrP^Sc^). Like PolyQ diseases and prion disease, the number of repeatable peptide units in the pathogenic protein influences the clinical outcome of individuals infected with *H. pylori* carrying Western CagA. In human proteins, however, repetitive unit lengths are mostly less than 10 amino acid residues, substantially shorter than the case of the *H. pylori* CagA EPIYA-C segment consisting of 34 amino acid residues. Expansion of a fairly long repetitive unit in effector molecules may be a unique strategy of pathogenic bacteria in making host-microbe interaction in favor of bacteria. In fact, *H. pylori* CagA is not the only bacterial effector containing expandable fragments with EPIYA or EPIYA-like tyrosine phosphorylation motifs. Eight additional bacterial EPIYA-like effectors have so far been reported^33^. As is the case of CagA, their repeat number may also determine the magnitude of effector functions in the target cells, which in turn influences clinical outcomes.

The present work provided a molecular insight into how CagA containing two or more (type II CagA), but not one (type I CagA), EPIYA-C segments substantially contributes to gastric carcinogenesis, thereby making infection with *H. pylori* carrying type II Western CagA a distinct clinical risk factor for gastric carcinoma.

## Methods

### Expression vectors

The pSP65SRα mammalian expression vectors for HA-tagged CagA-ABC and HA-tagged CagA-ABCCC have been described previously^12^,^39^. The pSP65SRα-CagA-ABCC vector was made by deletion of one EPIYA-C segment from pSP65SRα-CagA-ABCCC. The pSP65SRα-CagA-ABCCCCC and pSP65SRα-CagA-ABCCCCCCCC vectors were made by inserting one and two units of DNA sequences encoding triple-repeated EPIYA-C segments into pSP65SRα-CagA-ABCC, respectively. The pGEX6P2 *E. coli* expression vector for 6xHis-tagged CagA-ABCCC has been described previously^40^. The pGEX6P2-CagA(C3)-His was made by replacing the EPIYA motifs in the EPIYA-A and EPIYA-B segments with the EPIFA sequences, which cannot undergo tyrosine phosphorylation. The pGEX6P2-CagA(C1, C2, C5, or C8)-His vector was generated by replacing the DNA segment encoding the EPIYA-C segments in the pGEX6P2-CagA(C3)-His vector with that encoding the EPIYA-C segments in the pSP65SRα-CagA-ABC, -ABCC, -ABCCCCC, or -ABCCCCCCCC vector using *NaeI* and *Xba*I enzyme recognition sites. The gene encoding phosphorylation-resistant (PR) CagA (CagA-abccc), in which all the five EPIYA motifs was replaced by the EPIFA sequences, has been described previously^12^ and was cloned into pGEX6P2 vector to express recombinant PR-CagA in *E. coli*. The DNA sequence encoding the N-terminal region (residues 1–220) of human SHP2 that contains two wild-type SH2 domains (SHP2/SH2) or functionally dead SH2 domains (SHP2/SH2D; where D means “dead”) has been described previously^9^. The DNA fragment encoding full-length SHP2, SHP2/SH2 or SHP2/SH2D was inserted into the pGEX6P2 vector. The cDNA fragment encoding v-Src was cloned into the pACYCDuet1 bacterial expression vector.

### Antibodies

An anti-HA monoclonal antibody (3F10; Roche) was used as a primary antibody for immunoprecipitation, immunoblotting or immunostaining. Anti-His-tag monoclonal antibody (OGHis; Medical & Biological Laboratories), anti-SHP2 polyclonal antibody (C-18; Santa Cruz Biotechnology), anti-phosphotyrosine monoclonal antibody (4G10; Millipore), anti-actin polyclonal antibody (C-11; Santa Cruz Biotechnology), and anti-CagA polyclonal antibody (HPP-5003-9; AUSTRAL Biologicals) were used as primary antibodies for immunoblotting.

### Protein expression and purification

Expression and purification of recombinant CagA was performed as described previously^5^. Tyrosine phosphorylation of recombinant CagA was performed as follows. *E. coli* BL21(DE3) strain was co-transformed with pGEX6P2-CagA vector and pACYCDuet1-v-Src vector and cultured with Terrific-Broth at 37 °C. After reaching the OD_600_ = 1.0, protein expression was induced by 0.1 mM isopropyl-1-thio-β-D-galactopyranoside (IPTG) at 18 °C for overnight. Purification of tyrosine-phosphorylated (pY) CagA was performed by the method used for non-pY-CagA purification. In brief, bacteria were collected and re-suspended in Ni-binding buffer (20 mM Tris-HCl, pH 8.0, 100 mM MgCl_2_, 10 mM imidazole, 0.3 mg/ml benzamidine, 2 mM Na_3_VO_4_), and were lysed by sonication. Nickel-nitrilotriacetic acid (Ni-NTA) agarose beads (QIAGEN) were added to the soluble fraction and were incubated at 4 °C for 1 h. Beads were washed with Ni-wash buffer (20 mM Tris-HCl, pH 8.0, 100 mM MgCl_2_, 20 mM imidazole, 2 mM Na_3_VO_4_) and proteins bound to beads were eluted with Ni-elution buffer (20 mM Tris-HCl, pH 8.0, 100 mM MgCl_2_, 250 mM imidazole, 2 mM Na_3_VO_4_). The eluates were loaded onto Glutathione Sepharose 4B beads (GE Healthcare) and incubated at 4 °C for 1 h. Beads were then washed with GST-binding buffer (50 mM Tris-HCl, pH7.3, 150 mM NaCl, 5 mM EDTA, 2 mM DTT) and were re-suspended in GST-cleavage buffer (50 mM Tris-HCl, pH 7.0, 150 mM NaCl, 1 mM EDTA, 1 mM DTT). The GST-tags were cleaved with PreScission protease (GE Healthcare) at 4 °C for 16 h in GST-cleavage buffer, and the unbound fraction was collected. Buffer was changed to CagA-purification buffer (20 mM Tris-HCl, pH 8.0, 500 mM NaCl) using HiPrep 26/10 Desalting column (GE Healthcare). Proteins were applied to HisTrap HP column (GE Healthcare) and were washed with a linear gradient of 0–60 mM imidazole in CagA-purification buffer. Then, pY-CagA-His were eluted with CagA-purification buffer containing 250 mM imidazole. Finally, gel-filtration chromatography was performed using Superose 6 column (GE Healthcare) in CagA-purification buffer. Expression and purification of recombinant SHP2, SHP2/SH2, GST-SHP2/SH2, or GST-SHP2/SH2D was performed as follows. Bacteria were cultured in LB at 37 °C until the OD_600_ reaching 0.8. Proteins were induced with 0.1 mM IPTG for 16 h at 25 °C. The bacterial pellets were re-suspended with Equilibration buffer (25 mM Tris-HCl, pH 7.5, 150 mM NaCl, 5 mM EDTA, 10 mM β-mercaptoethanol) and were lysed by sonication. The soluble fraction was loaded onto Glutathione Sepharose 4B beads (GE Healthcare) and was washed with Wash buffer W1 (25 mM Tris-HCl, pH 7.5, 150 mM NaCl, 1% TritonX-100, 10 mM β-mercaptoethanol) and followed by Wash buffer W2 (25 mM Tris-HCl, pH 7.5, 150 mM NaCl, 10 mM β-mercaptoethanol). GST-tagged SHP2/SH2 or SHP2/SH2D was eluted with Wash buffer W2 containing 10 mM reduced glutathione.

### GST pull-down assay

For the GST pull-down assay, His-tagged recombinant CagA (at a final concentration of 50 nM) and GST-tagged SHP2/SH2 (at a final concentration of 50 nM) were mixed and incubated at 4 °C for 1 h. Glutathione Sepharose 4B beads were then added to the protein mixture and incubated at 4 °C for an additional 1 h. The beads were washed with wash buffer (50 mM Tris-HCl, pH 7.0, 150 mM NaCl, 1 mM EDTA, 1 mM DTT, 0.01% TritonX-100), and proteins bound to the beads were subjected to SDS-polyacrylamide gel electrophoresis (PAGE) and were visualized using 2D-Silver Stain Reagent II (COSMO BIO Co., ltd.) according to the manufacturer’s protocol.

### Surface plasmon resonance

Surface plasmon resonance was measured by using Biacore X100. Purified CagA proteins were immobilized on CM5 sensor chip with ammine coupling. Purified SHP2 or SHP2/SH2 was added to the flow cell at indicated concentrations. Dissociation constants were calculated by Scatchard plots using Biacore X100 Evaluation Software.

### Cell culture, transfection, and infection

AGS human gastric epithelial cells were cultured in RPMI 1640 medium supplemented with 10% fetal bovine serum (FBS) at 37 °C in 5% CO_2_. At 12 h after seeding, cells were transfected with expression vector by using Lipofectamine 2000 regent (Invitrogen) according to the manufacture’s protocol. *H. pylori* NCTC11637 strain was cultured as described previously^29^. At 12 h after seeding, AGS cells were infected with *H. pylori* for 5 h at a multiplicity of infection (MOI) of 100.

### Immunostaining

Cells were fixed with Mildform 10 N (Wako) for 20 min and were permiabilized with 0.5% Triton X-100 for 20 min at room temperature. The fixed cells were treated with a primary antibody and were visualized with Alexa Fluor 488-conjugated secondary antibody (Invitrogen). The nuclei were stained with 4′, 6-diamidino-2-phenylindole dihydrochloride n-hydrate (DAPI; Wako). Images were captured by confocal fluorescent microscopy (FV1200; OLYMPUS).

### Immunoblot and immunoprecipitation

At 24 h after transfection, AGS cells were harvested and lysed in lysis buffer (50 mM Tris-HCl, pH 7.5, 100 mM NaCl, 5 mM EDTA, 1% Brij-35, 2 mM Na_3_VO_4_, 10 mM NaF, 10 mM β-glycerophosphate, 10 μg/ml aprotinin, 10 μg/ml leupeptin, 10 μg/ml trypsin inhibitor, 2 mM PMSF). Total cell lysates and immunoprecipitates were subjected to SDS-PAGE and proteins were electro-blotted onto PVDF membrane filter. Protein transferred filters were incubated with primary antibodies and proteins were visualized by using Western Lightning Plus-ECL (Perkin Elmer, Inc.).

### Phos-tag SDS-PAGE analysis

Phos-tag SDS-PAGE was performed according to the method described previously^25^. In brief, protein samples were separated on a 5% polyacrylamide gel containing 15 mM Tris-HCl, pH 8.8, 18–20 μM Phos-tag (Wako Pure Chemical Industries), 36–40 μM MnCl_2_, and 0.1% SDS. After separation, proteins were electro-blotted onto a PVDF filter and were subjected to immunoblotting using an anti-CagA antibody or an anti-phosphotyrosine antibody.

### Cell invasion assay

Collagen gel solution was prepared by mixing Cellmatrix Type I-A (Nitta-Gelatin Inc.), serum-free 5xRPMI 1640 medium, and Reconstitution buffer (0.05 N NaOH, 2.2% NaHCO_3_, 20 mM HEPES) at a ratio of 7:2:1 on ice. One hundred μl of the chilled collagen mixture solution was then loaded onto the 24-well, 6.5 mm diameter, 8.0 μm pore-size Cell Culture Insert (Becton, Dickinson and Company), and was incubated for 1 h at 37 °C. At 4 h after transfection, cells were re-seeded onto the opposite side of cell culture insert containing collagen gel. Cells were attached for 4 h, and then cell culture insert was inverted. Cell culture insert was placed in serum free medium, and medium containing 10% serum and 30 ng/ml EGF was added to top of the gel. Cells were cultured for 5 days and were stained with Calcein-AM solution. Confocal x-y images observed every 5 μm from the surface of cell culture membrane by using confocal fluorescent microscopy. Images of invaded cells and non-invaded cells were captured and analyzed by using the ImageJ software (National Institutes of Health; imagej.nih.gov/ij/).

## Additional Information

**How to cite this article**: Nagase, L. *et al.* Dramatic increase in SHP2 binding activity of *Helicobacter pylori* Western CagA by EPIYA-C duplication: its implications in gastric carcinogenesis. *Sci. Rep.* **5**, 15749; doi: 10.1038/srep15749 (2015).

## Supplementary Material

Supplementary Information

Supplementary Movie s1

Supplementary Movie s2

Supplementary Movie s3
