# Supplementary material for: Dramatic increase in SHP2 binding activity of Helicobacter pylori Western CagA by EPIYA-C duplication: its implications in gastric carcinogenesis
Source: Sci Rep. 2015 Oct 28;5:15749. doi: 10.1038/srep15749 (PMC4623810; doi:10.1038/srep15749)
Supplement: Supplementary Information [file srep15749-s1.pdf]

Supplementary Information

Dramatic increase in SHP2 binding activity of *Helicobacter pylori* Western CagA by EPIYA-C duplication: its implications in gastric carcinogenesis

Lisa Nagase, Takeru Hayashi, Toshiya Senda, and Masanori Hatakeyama

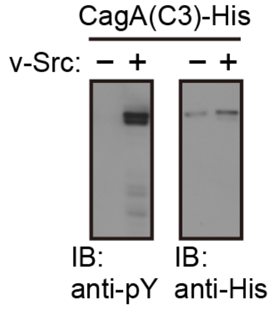

Supplementary Figure S1. Immunoblot analysis of recombinant CagA produced in *E. coli* with the use of an anti-phosphotyrosine antibody. The His-tagged CagA(C3) protein [CagA(C3)-His] produced in *E. coli* in the absence (-) or presence (+) of v-Src was subjected to immunoblotting (IB) with an anti-phosphotyrosine (pY) antibody or an anti-His antibody. Tyrosine phosphorylation signal was hardly detectable at the position corresponding to recombinant CagA(C3) made in *E. coli* without v-Src co-expression.

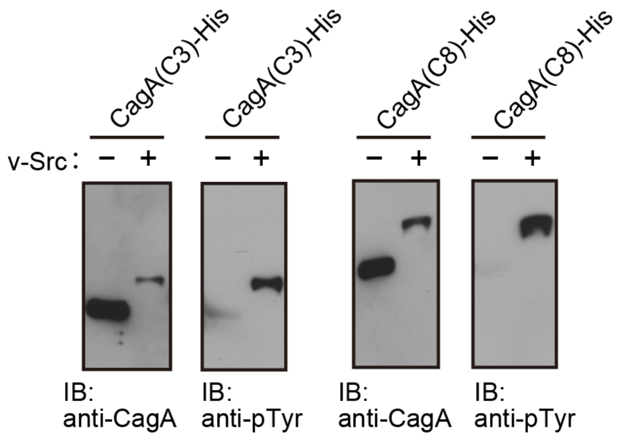

Supplementary Figure S2. Efficiency of CagA tyrosine phosphorylation by v-Src in *E. coli.* (*left two panels*) The His-tagged CagA(C3) proteins, purified from *E. coli* with or without v-Src expression, were separated on Phos-tag SDS-PAGE (5% polyacrylamide, 20 μM Phos-tag, 40 μM $MnCl_2$) and subjected to immunoblotting with the indicated antibodies. (*right two panels*) The CagA(C8)-His protein, purified from *E. coli* with or without v-Src expression, were separated on Phos-tag SDS-PAGE (5% polyacrylamide, 18 μM Phos-tag, 36 μM $MnCl_2$) and subjected to immunoblotting (IB) with the indicated antibodies.

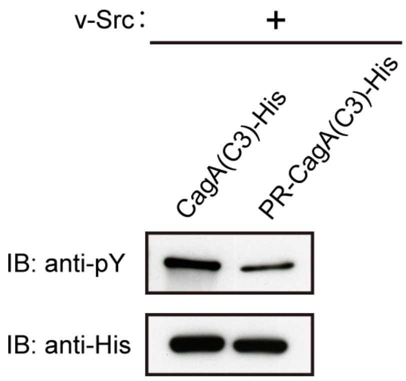

Supplementary Figure S3. Tyrosine phosphorylation status of v-Src-treated CagA in *E. coli*. CagA(C3)-His and PR-CagA(C3)-His prepared from *E. coli* expressing v-Src were subjected to immunoblotting with an anti-His antibody or an anti-phosphotyrosine (pY) antibody. IB: immunoblotting.

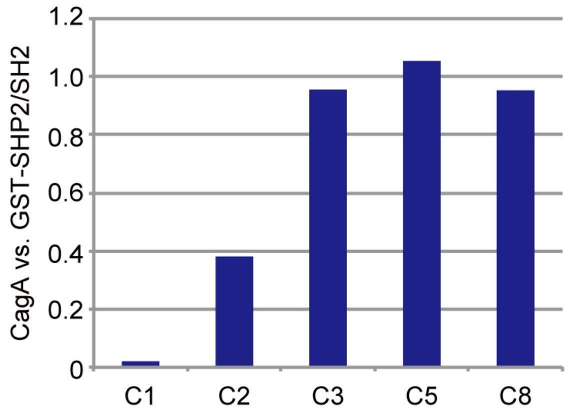

Supplementary Figure S4. Densitometric quantitation of CagA pulled down by GST-SHP2/SH2. Silver staining bands of GST-SHP2/SH2 and CagA(Cn)-His shown in Fig. 1c (lanes 8-12) were quantitated by densitometry. The relative amount of GST-SHP2/SH2-bound CagA was determined by dividing the value of the CagA band by the value of the GST-SHP2/SH2 band in each lane.

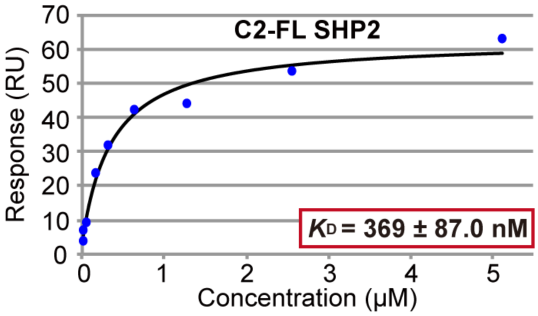

Supplementary Figure S5. SPR analysis of interaction between CagA and full-length SHP2. Surface plasmon resonance (SPR) analysis was performed using pY-CagA(C2) and full-length SHP2 (FL SHP2). Equilibrium dissociation constant (K_D) was determined by Scatchard plot analysis.

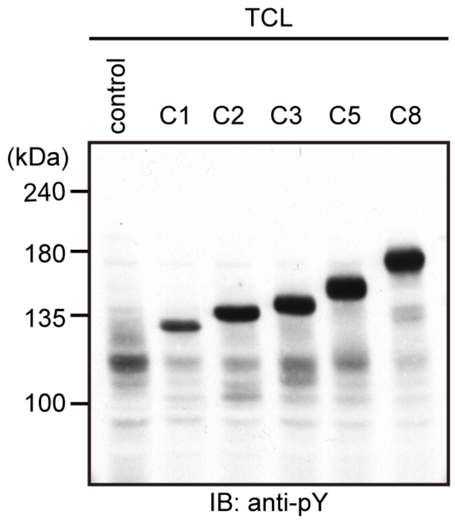

Supplementary Figure S6. *H. pylori* CagA is a major tyrosine-phosphorylated protein in AGS cells transiently transfected with a CagA(Cn) vector. AGS cells were transiently transfected with an HA-tagged Western CagA (C1, C2, C3, C5 or C8) vector. At 24 h after transfection, total cell lysates (TCLs) were prepared from the transfected AGS cells and were subjected to immunoblotting with an anti-phosphotyrosine (pY) antibody. The strongest band in each of lanes 2-6 was the CagA protein with a variable number of EPIYA-C segments. Of note, many tyrosine-phosphorylated endogenous proteins (some being smear-like) were also detectable on the immunoblot but were much less abundant than CagA.

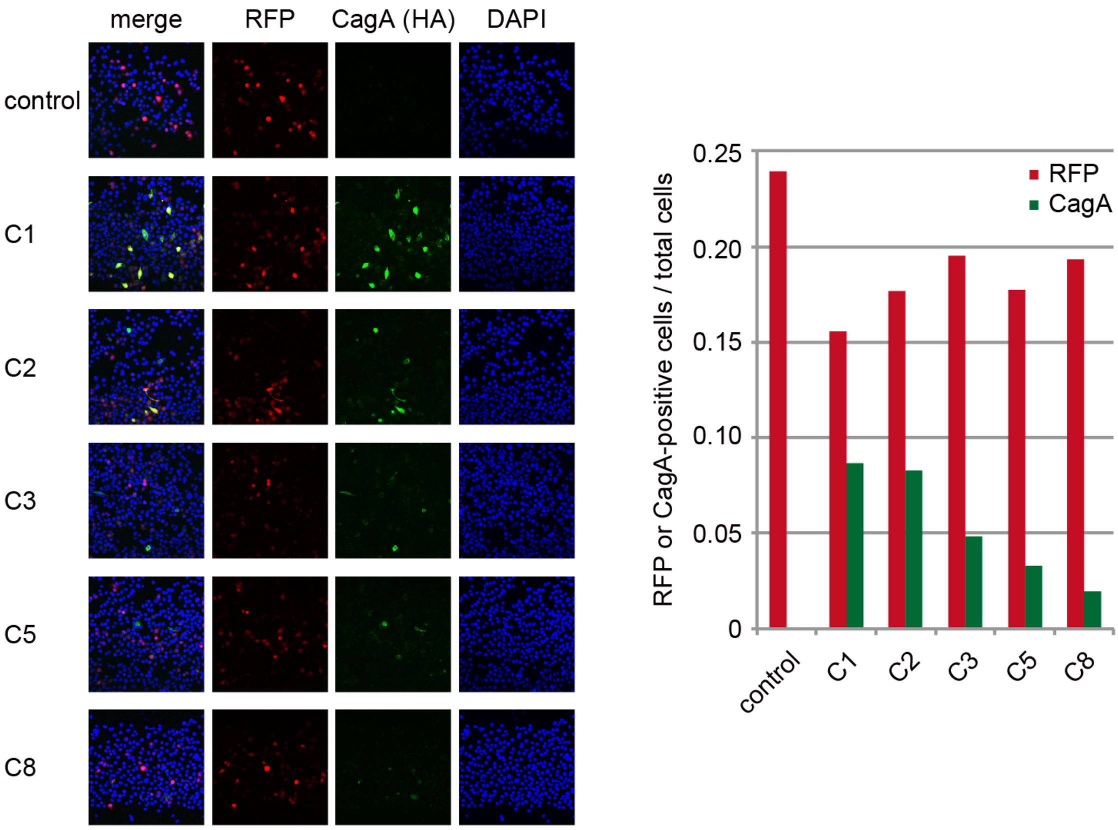

Supplementary Figure S7. CagA-positive cells decrease as the number of EPIYA-C segments increases in a transient transfection experiment. (*left*) AGS cells were transiently co-transfected with an HA-tagged Western CagA (C1, C2, C3, C5 or C8) vector and a red fluorescent protein (RFP) vector. At 24 h after transfection, expression of RFP (red) and HA-CagA (green; stained by an anti-HA antibody) was investigated by fluorescence microscopy. To count the total cell number, nuclei were also visualized by DAPI staining (blue). (*right*) The ratios of RFP-positive cells/total cells (red bars), which indicated transfection efficiency, and CagA-positive cells/total cells (green bars) presented in the left panels were calculated. The results of the experiment showed that transfection efficiencies as determined by RFP expression were comparable among the transfection groups, whereas CagA-positive cells were consistently reduced as the number of EPIYA-C segments increased.

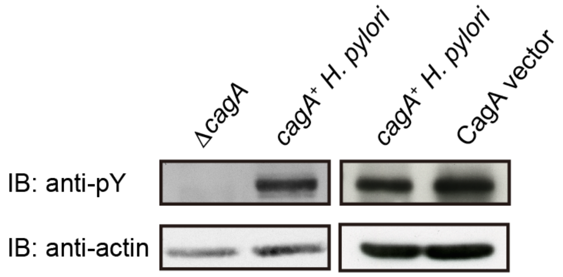

Supplementary Figure S8. Comparison of the levels of infected CagA and transfected CagA in AGS cells. (*left*) AGS cells were infected with *H. pylori* 11637 strain that produces the ABCCC-type Western CagA (CagA-ABCCC) or its isogenic strain lacking the *cagA* gene (*ΔcagA*) for 5 h. Total cell lysates were prepared and were then subjected to immunoblotting (IB) with an anti-phosphotyrosine (pY) antibody or an anti-actin antibody (for protein loading control). (*right*) AGS cells were infected with the *H. pylori* 11637 strain for 5 h or transiently transfected with a CagA vector that directs the expression of CagA-ABCCC for 24 h. Total cell lysates were prepared and were then subjected to immunoblotting (IB) with an anti-pY antibody or an anti-actin antibody (for protein loading control). It should be noted that total cell lysates prepared from *H. pylori*-infected AGS cells contain the CagA protein derived from the attached *H. pylori*, and the level of CagA delivered into the host cells cannot be determined by an anti-CagA antibody in the infection experiment. On the other hand, *H. pylori*-delivered CagA or transfected CagA undergoes tyrosine phosphorylation by Src and/or Abl kinases in the host AGS cells, indicating that the level of tyrosine-phosphorylated (pY) CagA reflects the amount of the CagA protein present in the host cells.

Supplementary Movies

Movie S1. Migration of control AGS cells. AGS cells were transiently transfected with a control vector. At 12 h after transfection, cells were observed by differential interference contrast microscopy at 3 min intervals for 6 h.

Movie S2. Migration of AGS cells expressing CagA with a single EPIYA-segment. AGS cells were transfected with a mammalian expression vector for an HA-tagged ABC-type Western CagA (CagA-ABC). At 12 h after transfection, cells were observed by differential interference contrast microscopy at 3 min intervals for 6 h.

Movie S3. Migration of AGS cells expressing CagA with two EPIYA-C segments. AGS cells were transfected with a mammalian expression vector for an HA-tagged ABCC-type Western CagA (CagA-ABCC). At 12 h after transfection, cells were observed by differential interference contrast microscopy at 3 min intervals for 6 h.
